# Optimized pathogen reduction of double‐dose platelets pooled from seven buffy coats using the TACSI® system

**DOI:** 10.1111/vox.70091

**Published:** 2025-08-19

**Authors:** Alice Lorusso, Liam Morgan, Aileen Farrelly, Mark Lambert, Barry Doyle, Paul Hendrick, Einas Elsheikh, Andrew Godfrey, Tor Hervig, Allison Waters

**Affiliations:** ^1^ Irish Blood Transfusion Service Dublin 8 Ireland; ^2^ School of Medicine Trinity College Dublin Dublin 2 Ireland; ^3^ Department of Clinical Science University of Bergen Bergen Norway; ^4^ Physiotherapy and Sports Science University College Dublin School of Public Health Dublin Ireland

**Keywords:** buffy coats, INTERCEPT, pathogen inactivation, pathogen reduction, pooled platelets, TACSI® system

## Abstract

**Background and Objectives:**

Pathogen reduction technologies (PRTs) can enhance platelet safety by mitigating pathogenic contamination risks. This study describes, for the first time, the in vitro platelet quality and function assessment of whole blood–derived double‐dose (DD) pools, prepared using seven buffy coats (BCs) pooled on the Terumo Automated Centrifuge & Separator Integration (TACSI)® system and pathogen‐reduced (PR) with the INTERCEPT™ Blood System (IBS).

**Materials and Methods:**

DD platelet pools were prepared by pooling seven ABO‐identical BCs with 280 mL of additive solution platelet additive solution E using the TACSI® system. Process optimization ensured compliance with the European Directorate for the Quality of Medicines (EDQM) guidelines and IBS entry specifications. Units were split and either treated with IBS or left untreated, and stored agitated in INTERCEPT storage bags at 22°C for 8 days. Platelet quality was assessed at baseline and at Days 2, 6 and 8 using quality control tests and flow cytometry.

**Results:**

All units complied with EDQM specifications. Platelet concentration significantly decreased in IBS‐treated platelets (*p* < 0.05). Both IBS‐treated and untreated platelet units exhibited similar stability for mean platelet volume (MPV), gas exchange and pH. Higher glucose values were detected in IBS‐treated platelets. CD62P expression and phosphatidylserine (PS) exposure significantly increased over storage in both groups, indicating comparable storage lesion and apoptosis, respectively. No differences were observed in the pro‐coagulant activity of platelets, as both groups were responsive to agonist stimulation.

**Conclusion:**

The findings support the use of the IBS for pathogen reduction in larger platelet pools and highlight the system's effectiveness in maintaining platelet functionality for transfusion purposes.

**Highlights**

- This is the first description of the preparation of double‐dose platelet concentrates from pools of seven buffy coats (BCs) using the Terumo Automated Centrifuge & Separator Integration (TACSI)® system, treated using the INTERCEPT™ Blood System (IBS) pathogen reduction technology.
- Platelet quality and functionality are adequately maintained following IBS treatment.
- Pathogen‐reduced BCs pooled using the TACSI® system comply with the European Directorate for the Quality of Medicines (EDQM) and Healthcare Guidelines.

## INTRODUCTION

Prevention of transfusion‐transmitted infections (TTIs), including screening for bacterial contamination, is an ongoing concern in transfusion medicine [1, 2]. Platelets carry a greater risk of bacterial contamination due to their storage at room temperature, which can lead to TTIs, especially in immunocompromised patients [2]. To enhance transfusion safety, blood establishments worldwide have adopted pathogen reduction (PR) technologies such as the INTERCEPT Blood System (IBS), which inactivates a broad spectrum of viruses, bacteria and protozoa, as well as residual donor leukocytes, thus reducing the risk of transfusion‐associated graft‐versus‐host disease (TA‐GVHD) [3, 4, 5, 6].

The INTERCEPT system is a PR technology that uses amotosalen, a psoralen‐based photoactive compound, combined with ultraviolet A (UVA) light to inactivate pathogens while preserving platelet functionality [4, 7]. During pathogen inactivation, platelets are mixed with a defined concentration of amotosalen, which intercalates into DNA and RNA, and, upon UVA exposure, forms crosslinks between pyrimidine bases, preventing replication and transcription [7]. Following light treatment, an integrated compound adsorption device (CAD) removes residual amotosalen and unbound photoproducts, ensuring minimal residual levels and making the final platelet product safe for transfusion [7, 8].

Several blood services worldwide have incorporated the IBS into their routine blood processing workflows to enhance transfusion safety and reduce the risk of TTIs [3, 9]. However, the operational and economic efficiency of implementing pathogen inactivation technologies in routine blood banking remains a challenge, particularly when processing large volumes of blood components while maintaining platelet quality post‐processing and during storage [10, 11]. To address these challenges, some blood services have started to use pooled buffy coats (BCs) to produce double‐dose (DD) platelet units suitable for pathogen reduction [10, 11, 12]. This approach balances operational efficiency and cost while also mitigating the risks associated with exposure to multiple donors [10, 11, 12].

Traditionally, whole blood (WB)–derived platelet concentrates (PCs) are prepared by pooling four to six BCs to produce single‐dose (SD) PCs [13, 14]. More recently, seven or eight BCs have been pooled to generate DD platelet units, which can then be split into two standard therapeutic doses [10, 11, 12, 15]. This method maximizes the use of donated WB units while reducing processing workload, improving inventory management and ensuring compliance with regulatory standards [10, 11]. However, a notable limitation is the lack of validated automated systems for platelet pooling and leukoreduction that are compatible with this approach [16].

The aim of the present study was to address this challenge by preparing seven BCs pooled using the Terumo Automated Centrifuge & Separator Integration (TACSI) system and assess, for the first time, its compatibility with PR‐treated DD pools as well as any impact on in vitro platelet quality.

## MATERIALS AND METHODS

### WB collection and BC preparation

This study was carried out as part of the Irish Blood Transfusion Service product development, so ethical approval was not sought. To comply with the IBS requirements for PR, BCs and pooled products were required to meet the pre‐processing specifications, which were defined by the manufacturer (Cerus Corp., USA) based on internal validation studies and in alignment with European Directorate for the Quality of Medicines (EDQM) guidelines.

WB units (~470 mL ± 10%) were collected from healthy volunteer donors using top‐and‐bottom quadruple blood bag systems (Macopharma, France). Immediately after collection, the units were placed on 1,4‐butanediol phase‐change cooling plates (Compocool, Fresenius HemoCare GmbH, Germany) and stored overnight at ambient temperature. On Day 1, the WB units were centrifuged at 3500 rpm for 12 min at 22 ± 2°C using a Roto Silenta 630 RS centrifuge (Andreas Hettich GmbH & Co. KG, Germany). Automated component separation into plasma, red cell concentrates (RCCs) and BCs was performed using a Compomat G5 (Fresenius Kabi, Germany) separator operating under an optimized program, specifically designed to produce small‐volume BCs suitable for DD platelet pool production. Target INTERCEPT specifications for BCs included the following: volume 45 ± 2 mL; haematocrit (Hct) 36% ± 2%; red blood cell content 16 ± 2 mL; plasma volume 29 ± 2 mL; minimum platelet count of ≥90 × 10^9^/unit. BCs were segregated by ABO/RhD and CMV status and stored for at least 2 h at 22 ± 2°C prior to pooling.

### TACSI® PL pooling optimization

**FIGURE 1:**
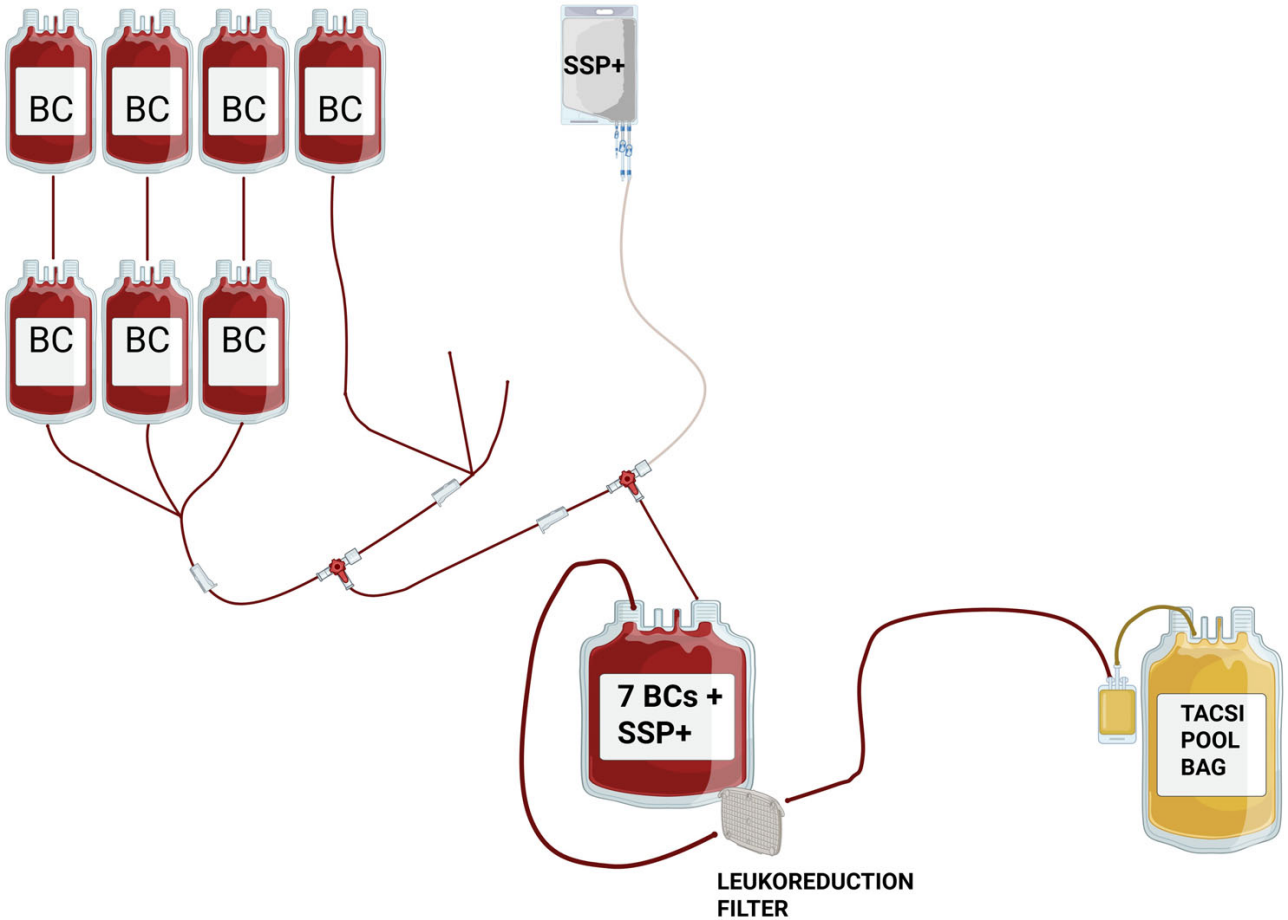
Schematic representation of seven buffy coats connected to the Terumo Automated Centrifuge & Separator Integration (TACSI) pooling set and SSP+ additive solution. Seven ABO‐identical buffy coats (BCs) are sterilely attached in a train configuration using a sterile connection device to a closed TACSI® PL pooling set, which includes (i) an intermediate pooling bag for manual mixing, (ii) a pre‐attached leukoreduction filter, (iii) a final storage bag labelled TACSI POOL BAG, (iv) six satellite lines for BC connection and (v) a dedicated line for platelet additive solution (PAS) SSP+. The seven BCs and 280 mL of SSP+ are manually mixed (10 inversions) and transferred into the intermediate pooling bag. The system is then mounted on the TACSI® device for automated processing, including centrifugation, sedimentation, leukoreduction and transfer of the platelets in PAS into the final storage bag.

Seven ABO‐identical BCs were connected to 280 mL of platelet additive solution (PAS, SSP+; MacoPharma, France) and the TACSI bag system (Terumo BCT, USA) using a sterile connecting device (TSCD®‐II, Terumo, USA), following the train configuration illustrated in Figure 1. The combined BCs and PAS were manually mixed (10 inversions) into an intermediate pooling bag (part of the closed TACSI system kit and pre‐attached to a leukoreduction filter), which was then mounted on the TACSI device for automated processing. The pooled product (manual mix of seven BCs and SSP) was tested and required to meet the following pre‐processing specifications: volume 600 ± 20 mL; Hct ≤ 20%; platelets (PLTs) > 600 × 10^9^; plasma ratio 32%–47%.

TACSI® processing includes centrifugation and sedimentation of blood components, optical separation, leukoreduction via a pre‐attached filter and final transfer of the platelet‐rich fluid into the storage bag. To accommodate larger pool production, the program was optimized based on the manufacturer's recommendations by adjusting key parameters that control each phase. This included increasing the detector sensitivity level to better identify the interface between the platelet‐rich fluid and the cellular layer (red cells, white cells and residual platelets); improving flow control during filtration; and reducing platelet loss. The Wait Integral parameter, which controls how long the system waits before starting the separation step, was reduced to optimize sedimentation timing for seven BC pools. These adjustments improved platelet recovery, filter clearance and overall processing efficiency.

A total of 10 DD pools were initially prepared and assessed to confirm compliance with INTERCEPT PR entry criteria, which included a final volume of 375–420 mL, platelet content ≥450 × 10^9^/unit, recovery ≥75%, absence of visible erythrocytes (visual assessment), swirling score 1–3, aggregate score ≤1, minimum volume of ≥35 mL per 60 × 10^9^ platelets, pH ≥6.4 at the end of shelf‐life and plasma ratio 32%–47%.

Subsequently, 12 additional DD pools were prepared and treated with INTERCEPT. These were split into 24 SD PR‐treated platelet components, which were sampled after CAD on Day 2 and at expiry (Day 8) for full quality control (QC) assessment. To evaluate the impact of CAD incubation time, 12 PR splits were incubated for 16 h (maximum recommended duration) and the remaining 12 for 6 h (minimum), all maintained at 22 ± 2°C under continuous agitation.

### Platelet storage study design

**FIGURE 2:**
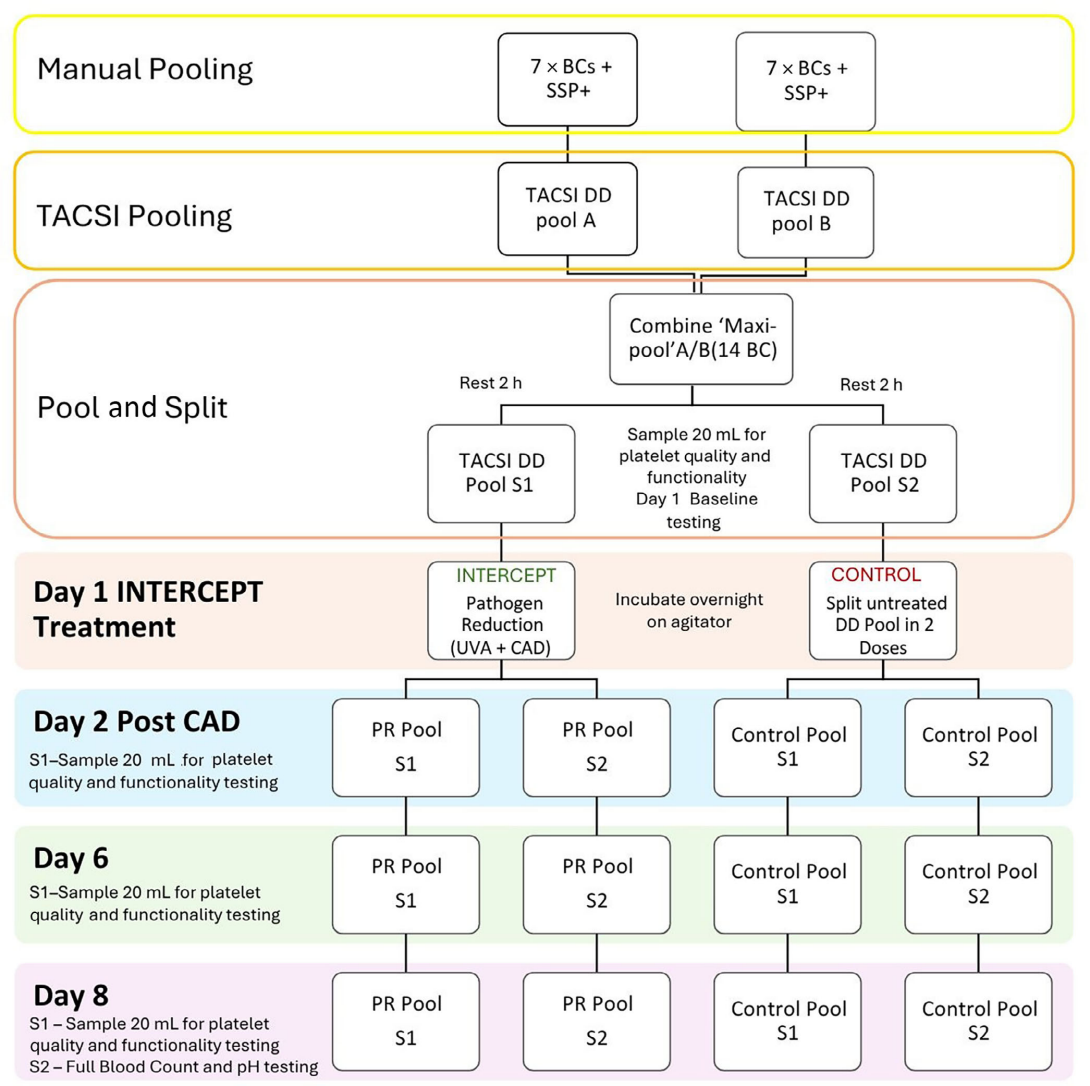
Schematic representation of the pool‐and‐split method used to evaluate 8‐day storage of pathogen‐reduced whole blood platelets (WB‐PLTs) (double‐dose [DD]). The figure illustrates two representative DD pools (out of 10 total). In the first swim lane, two pools are manually prepared by mixing seven buffy coats (BCs) and 280 mL platelet additive solution SSP+. The second lane shows the automated production of two DD pools (A and B) using the Terumo Automated Centrifuge & Separator Integration (TACSI)® system. The third swim lane shows the pooling of the two DD pools into one maxi‐pool (A/B), which is then split into two matched units: S1, assigned to the INTERCEPT™ pathogen reduction (PR) arm, and S2, serving as the untreated control. Baseline samples were collected from both S1 and S2 prior to PR treatment (Day 1). CAD, compound adsorption device; S1, Split 1 (PR‐treated); S2, Split 2 (untreated control). UVA, ultraviolet A.

To assess the effect of PR on platelet quality and function during storage, a paired ‘pool‐and‐split’ study was designed (Figure 2). A total of 10 DD pools were prepared, each from seven ABO‐identical BCs and 280 mL SSP+ and processed using the TACSI® system. To minimize donor‐related variability, two DD pools were combined to form five larger maxi‐pools (14 BCs each). After resting (2 h), each maxi‐pool was split into two identical DD units (*n* = 10). One split (S1) was assigned to PR test arm, while the other split (S2) served as an untreated control, creating five matched PR‐control pairs. Baseline samples were collected from both S1 and S2 on Day 1 prior to PR treatment. Following treatment, all components were divided into SD units and stored in INTERCEPT platelet storage bags (Cerus, USA, ref. PN INT823) at 22 ± 2°C under continuous agitation in a flat‐bed, temperature‐controlled agitator (Helmer scientific, USA) for up to 8 days. Platelet quality and function were assessed at Days 1 (baseline, pre‐treatment), 2, 6 and 8. To avoid potential confounding effects of repeated sampling, S2 of each treated and control unit was reserved for testing at expiry only (Day 8) to assess platelet quality at the end of storage.

### In vitro quality and functional testing

Platelet count, mean platelet volume (MPV) and residual leucocyte content were measured using a Sysmex XN‐1000™ haematology analyser (Sysmex Corporation, Japan). Metabolic parameters (pH, pO_2_, pCO_2_) were assessed using the Roche Omni S blood gas analyser (Roche Diagnostics, Switzerland). Glucose concentration was quantified using the Cobas c311 automated analyser (Roche Diagnostics, Switzerland). Visual swirling was scored (0 = none, 3 = optimal), and the presence of visible aggregates was recorded.

#### Platelet activation (CD62P expression)

Platelet activation was evaluated by flow cytometry (FACSCanto™ II, BD Bioscience, USA). Surface expression of P‐selectin (CD62P) was qualified at baseline and following stimulation with the thrombin receptor‐activating peptide (TRAP; Sigma‐Aldrich, USA). Pathogen‐reduced platelet concentrates (PR‐PLTs) were adjusted to a final concentration of 200 × 10^6^ platelets/mL and incubated with 4.4 μL of a 5 mM TRAP stock solution, diluted in HEPES‐buffered saline to a final concentration of 40 μM. Samples were fixed with 1% CellFIX™ (BD Biosciences, USA) to arrest further activation and stained with fluorescein isothiocyanate (FITC)‐conjugated anti‐CD62P monoclonal antibody (BD Biosciences, USA) to identify activated platelets. A negative control using FITC‐conjugated immunoglobulin‐G isotype antibody (BD Biosciences, USA) was included with each sample to assess background fluorescence and non‐specific binding. Flow cytometric analysis was performed immediately, under low flow rate conditions.

#### Platelet apoptosis (PS exposure)

Phosphatidylserine (PS) exposure was assessed using Annexin V‐FITC staining (BD Biosciences, USA). PR‐PLT samples were washed with calcium‐free phosphate‐buffered saline (PBS) and centrifuged at 500*g* for 5 min (Diacent‐12, Bio‐Rad Laboratories, USA). Pellets were resuspended in Annexin V binding buffer (BD Biosciences, USA). For each test, 10,000 platelet events were acquired using a FACSCanto II flow cytometer (BD Biosciences, USA). Positive control samples were treated with a calcium ionophore (Sigma‐Aldrich, USA) to induce maximum PS exposure, while negative control samples were suspended in calcium‐free PBS. All samples were incubated with FITC‐conjugated Annexin V and analysed by flow cytometry within 1 h of staining. Flow cytometric analysis was performed under medium flow rate conditions.

### Bacterial contamination testing

At expiry (Day 8), all PR‐treated and control platelet units were tested for bacterial contamination using the BacT/ALERT® 3D microbial detection system (bioMérieux, France). Aseptically collected samples (8 mL) were inoculated into both aerobic (BPA) and anaerobic (BPN) culture bottles. All bottles were incubated in the BacT/ALERT® 3D system for a maximum of 7 days.

### Statistical analysis

Statistical analyses were conducted using IBM SPSS Statistics v25.0 (IBM Corp., USA). Data were managed and graphed using Microsoft Excel (Microsoft Corp., USA). Given the small sample size (*n* = 10) and non‐normal distribution of variables, non‐parametric methods were applied. The Wilcoxon signed‐rank test was used for paired comparisons between PR‐treated and control units. Repeated measures across storage days were analysed using Friedman's test. Statistical significance was defined as *p* <0.05.

## RESULTS

### Process optimization

All parameters assessed during individual BC validation following program optimization met the required INTERCEPT™ specifications. Pools (*n* = 10) of seven ABO‐identical BCs and 280 mL of SSP+ were all within the entry criteria before automated TACSI pooling. DD platelet pools (*n* = 10) prepared during TACSI® optimization met all INTERCEPT™ and EDQM entry specifications [17], with a mean volume of 414 ± 4 mL, platelet content of 558 ± 92 × 10^9^ per DD unit and a platelet concentration of 1348 ± 214 × 10^9^/L, equivalent to 46 ± 7 mL per 60 × 10^9^ platelets, in line with the Irish Blood Tranfusion Services (IBTS) and EDQM validation specifications. Platelet recovery was 90% ± 1.6%, and leucocyte content was 0.11 ± 0.05 × 10^6^/pool. The mean pH was 6.9 ± 0.1, and the plasma ratio averaged 40% ± 0.8%. All units showed optimal swirling (score 2 ± 0.5), no visible aggregates and no red cell contamination (Table 1). Residuals from TACSI processing (*n* = 10) showed effective separation with all parameters tested within optimization targets (residual volume <200 mL, Hct ≥ 60%, residual PLTs < 100 × 10^9^).

**TABLE 1:** Optimization and process validation of double‐dose pools of seven buffy coats stored in 280 mL SSP+ and pathogen reduced with the INTERCEPT™ Blood System.

Parameter	INTERCEPT input requirement^a^/EDQM specification^b^	Double‐dose pool (*n* = 10 DD)	Final INTERCEPT‐treated platelets (*n* = 24 SD)
Volume (mL)	375–420 (DD)^a^/≥175 (SD)^a^	414 ± 4	191 ± 6
Platelet content (×10^9^/unit)	>450 (DD)^a^/≥200 (SD)^b^	558 ± 92	243 ± 26
Platelet concentration (10^9^ cells/L)	N/A	1348 ± 214	1272 ± 142
Plasma ratio (%)	32%–47%^a^	40 ± 0.8	N/A
Red blood cell contamination (×10^9^/L)	<4^b^	Visually absent	N/A
Platelet recovery (%)	>80%^a^	90 ± 1.6	N/A
Volume per 60 × 10^9^ platelets (mL)	≥ 35^a^, ^c^/>40^2^	46 ± 7	48 ± 5
Leukocyte content (×10^6^/pool)	<1 per split pool^b^	0.11 ± 0.05	0.03 ± 0.0
Glucose (end of shelf‐life)	Above LOQ^b^	N/A	1.9 ± 0.9
pH (end of shelf‐life)	≥6.4^b^	6.9 ± 0.1	7.19 ± 0
Swirling	Score 1–3^c^	2 ± 0.5	3
Aggregates	Score ≤1^c^	0	0
Bacterial growth	Negative^b^	N/A	Negative

*Note*: Values are expressed as mean ± standard deviation unless otherwise indicated.

Abbreviations: DD, double‐dose; EDQM, European Directorate for the Quality of Medicines; LOQ, limit of quantification; N/A, not applicable; SD, single‐dose.

^a^ INTERCEPT™ Blood System input requirement (met by ≥90% of tested units).

^b^ EDQM specification (met by ≥90% of tested units).

^c^ Internal Irish Blood Tranfusion Service (IBTS) specification.

The 12 DD platelet pools that underwent PR using the IBS had a mean volume of 403 ± 10 mL. Following treatment, the 24 resulting PR SD units exhibited a mean volume of 191 ± 6 mL, a platelet content of 243 ± 26 × 10^9^ per unit and a mean platelet concentration of 1272 ± 142 × 10^9^/L, equivalent to a minimum volume of 48 ± 5 mL per 60 × 10^9^ platelets (as per IBTS/EDQM validation specification). Leucocyte content was 0.03 ± 0.0 × 10^6^/unit. At the end of storage (Day 8), the mean pH was 7.19 ± 0.0 and glucose concentration was 1.9 ± 0.9 mmol/L. All units showed optimal swirling (score 3), with no visible aggregates or microbial contamination. All tested parameters complied with INTERCEPT and EDQM quality specifications (Table 1).

The effect of CAD incubation time is presented in Table 2. A higher mean platelet count was recorded following 16‐h incubation compared to 6‐h incubation.

**TABLE 2:** Descriptive comparison of double‐dose pathogen‐reduced platelet quality parameters after 6‐h and 16‐h compound adsorption device incubation.

Parameter	16‐h CAD incubation (*n* = 12)	6‐h CAD incubation (*n* = 12)
DD pool volume (mL)	401 ± 6	404 ± 14
Split volume (mL)	191 ± 4	192 ± 7
Platelet count (×10^9^/unit)	258 ± 26	227 ± 13
Volume per 60 × 10^9^ platelets (mL)	45 ± 4	51 ± 4
White blood cell count (×10^6^/unit)	0.04 ± 0.02	0.03 ± 0.02
pH at 22°C (end of shelf‐life)	7.17 ± 0.11	7.21 ± 0.06
Glucose (mmol/L, end of shelf‐life)	1.7 ± 0.8	2.03 ± 0.9

*Note*: Data are presented as mean ± standard deviation (SD).

Abbreviations: CAD, compound adsorption device; DD, double‐dose.

No significant differences were observed between groups in split volume or residual WBC content. pH values at Day 8 were maintained above the minimum required threshold in both groups, and glucose concentrations remained within acceptable ranges. No evidence of bacterial contamination or visible aggregates during storage was observed. All units presented excellent swirling.

### In vitro storage study

#### Platelet quality parameters

Platelet concentration in the PR‐treated platelets dropped significantly during storage (*p* = 0.017), while the control group remained stable (*p* = 0.323). Therefore, PR units had significantly lower platelet counts compared to the untreated controls at each time point (Table 3). MPV remained steady in both groups across all days, suggesting that platelets maintained their size and shape during storage. All platelet units showed good swirling throughout 8 days of storage, with a higher swirling score on Day 2 for PR platelets, confirming preserved morphological quality in both PR units and controls. No aggregates were observed in any of the PCs. Glucose consumption increased in both groups over time, but the PR‐treated platelets consistently had higher glucose reserves, indicating reduced metabolic activity compared to untreated units. The difference was significant on Days 2 and 6 (*p* = 0.043, Table 3). The pH values remained above 7.0 in both groups throughout storage. Oxygen and carbon dioxide levels (pO_2_ and pCO_2_) were similar in both groups, except for a small but significant drop in pCO_2_ in the PR units on Day 8 (1.9 vs. 2.1 kPa, *p* = 0.043), which may reflect slightly reduced metabolism.

**TABLE 3:** Pathogen‐reduced and conventional untreated platelet quality and functional parameters throughout 8 days of storage.

Platelet parameter	Day of storage	PR, mean ± SD	Repeated measure, *p*‐value	Control, mean ± SD	Repeated measure, *p*‐value	Paired analysis, *p‐*value
Platelet concentration (10^9^ cells/L)	Baseline	1282 ± 87	0.017*	1272 ± 87	0.323	0.343
	2	1230 ± 59		1277 ± 87		0.043*
	6	1176 ± 130		1285 ± 107		0.042*
	8	1143 ± 103		1238 ± 133		0.043*
MPV (fL)	Baseline	10.1 ± 0.2	0.824	10.2 ± 0.3	0.716	0.581
	2	10.2 ± 0.3		10.1 ± 0.3		0.257
	6	10.1 ± 0.3		10.1 ± 0.2		0.498
	8	10.1 ± 0.3		10.0 ± 0.3		0.109
Glucose (mmol/L)	Baseline	9.0 ± 0.4	0.002*	9.0 ± 0.3	0.002*	1.000
	2	8.1 ± 0.3		7.2 ± 0.6		0.043*
	6	4.6 ± 0.4		4.0 ± 0.7		0.043*
	8	2.2 ± 0.4		1.7 ± 0.5		0.068
pH (37°C) (kPa)	Baseline	7.1 ± 0.1	0.029*	7.1 ± 0.1	0.037*	0.317
	2	7.0 ± 0.1		7.1 ± 0.1		0.564
	6	7.1 ± 0.0		7.2 ± 0.0		0.083
	8	7.0 ± 0.1		7.1 ± 0.0		0.025*
pCO_2_ (37°C) (kPa)	Baseline	4.3 ± 0.5	0.003*	4.1 ± 0.6	0.006*	0.066
	2	4.0 ± 0.2		3.9 ± 0.7		0.893
	6	2.0 ± 02		2.2 ± 0.2		0.345
	8	1.9 ± 0.2		2.1 ± 0.1		0.043*
pO_2_ (37°C) (kPa)	Baseline	15.6 ± 6	0.043*	16.4 ± 6	0.290	0.893
	2	17.3 ± 5		21.6 ± 7		0.686
	6	21.8 ± 3		21.2 ± 3		0.345
	8	19.9 ± 4		19.0 ± 5		0.141
CD62P expression (%)	Baseline	27.1 ± 3	0.002*	29.1 ± 3	0.002*	0.043*
	2	30.5 ± 2		32.9 ± 2		0.043*
	6	42.2 ± 4		39.4 ± 3		0.043*
	8	47.1 ± 5		44.3 ± 4		0.080
Responsiveness to TRAP (%)	Baseline	83.5 ± 9	0.564	87.9 ± 5	0.896	0.043*
	2	83.9 ± 7		89.2 ± 2		0.043*
	6	83.1 ± 4		87.9 ± 4		0.043*
	8	86.2 ± 2		89.1 ± 3		0.043*
PS exposure (%)	Baseline	2.2 ± 1	0.006*	2.5 ± 1	0.004*	0.588
	2	2.6 ± 1		2.5 ± 1		1.000
	6	8.3 ± 3		6.7 ± 2		0.345
	8	11.1 ± 4		8.4 ± 1		0.080
Agonist‐induced PS exposure (%)	Baseline	99.4 ± 1	0.137	99.3 ± 1	0.017*	1.000
	2	99.5 ± 0.4		99.5 ± 0.3		0.157
	6	99.7 ± 0.3		99.8 ± 0.3		0.157
	8	99.6 ± 1		99.8 ± 0.3		0.197

*Note*: Mean ± standard deviation (SD) values are shown for each parameter measured at baseline, Days 2, 6 and 8 for PR and untreated control platelet components. Repeated measure *p‐*values refer to results from the Friedman test, which evaluated changes within groups over time. Paired analysis *p‐*values are from Wilcoxon signed‐rank tests comparing PR versus control platelets at each time point. Statistically significant values are indicated with an asterisk (*p <* 0.05).

Abbreviations: MPV, mean platelet volume; PR, pathogen‐reduced; PS, phosphatidylserine; SD, standard deviation; TRAP, thrombin receptor‐activating peptide.

#### Platelet functional parameters

**FIGURE 3:**
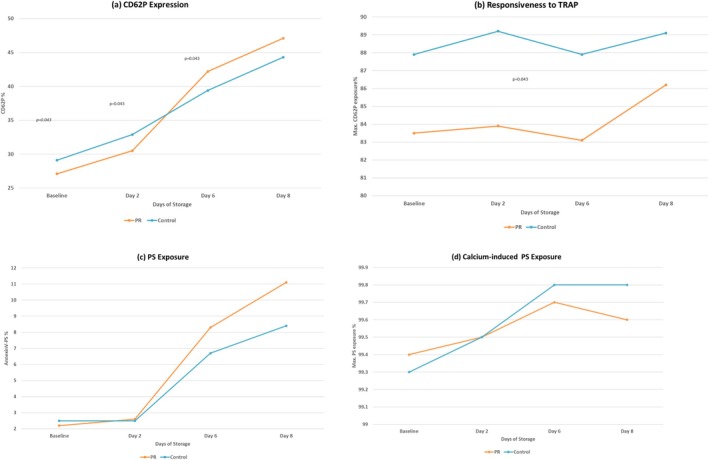
Functionality of pathogen reduction (PR)‐treated and control platelet components over 8 days of storage. (a) CD62P expression (%) increased significantly over storage in both groups (Friedman's test, *p* = 0.002). PR‐treated platelets showed a pronounced increase by Day 6. Statistically significant differences between PR‐treated and control units were observed at baseline, Days 2 and 6 (*p* = 0.043 for each, Wilcoxon signed‐rank test). Data represent mean ± SD (*n* = 5). (b) Responsiveness to thrombin receptor‐activating peptide (TRAP) stimulation remained consistently high (>80%) in both PR‐treated and control units throughout the 8‐day storage. Despite stable trends, PR‐treated platelets exhibited significantly lower TRAP‐induced activation at all time points (*p* = 0.043, Wilcoxon signed‐rank test). No significant time effect was observed (Friedman's test, *p* > 0.05). Data are shown as mean ± SD (*n* = 5). (c) Phosphatidylserine (PS) exposure (%) increased significantly during storage in both PR‐treated and control platelets (Friedman's test, *p* < 0.006). Although PR‐treated units showed numerically higher PS levels by Days 6 and 8, no statistically significant differences were observed between the groups at any time point (Wilcoxon signed‐rank test, *p* >0.05). Data are presented as mean ± SD (*n* = 5). (d) Maximum PS expression following calcium ionophore stimulation remained high (>99%) and stable in both groups. No significant differences were observed between PR‐treated and untreated platelets at any time point (Wilcoxon test, *p* >0.05). Friedman's test showed no time effect in controls but a slight variation in PR‐treated units (*p* = 0.017). Values represent mean ± SD (*n* = 5).

CD62P expression increased significantly throughout storage in both groups (*p* = 0.002), reflecting the progressive activation of platelets during storage. Paired analysis showed comparable activation trends between PR‐treated and untreated units, although CD62P levels were significantly higher in the untreated group at baseline (*p* = 0.043) and Day 2 (*p* = 0.043), while levels increased more prominently in the PR group towards the end of storage Day 6 (*p* = 0.043) (Table 3, Figure 3). Platelet responsiveness to TRAP stimulation remained consistently high in both groups, with values above 80% across all time points (*p* > 0.05), indicating preserved platelet reactivity. However, a modest but statistically significant reduction in TRAP‐induced activation was seen in PR‐treated platelets compared to controls at all time points (*p* = 0.043). PS externalization, a marker of apoptosis, also increased during storage. A significant change over time was seen in both groups (*p* < 0.006), with higher levels of PS exposure in PR‐treated platelets by Days 6 and 8. Calcium‐induced PS exposure remained high and stable in both groups and there were no differences between PR‐treated and untreated units at any time point (*p* > 0.5), indicating that the response to external stimulation was preserved following PR (Figure 3).

## DISCUSSION

PR remains a cornerstone of transfusion safety, particularly for platelet components stored at room temperature, which are susceptible to bacterial proliferation and may lead to TTIs [2, 18, 19]. IBS has been widely adopted in blood transfusion services to mitigate these risks while preserving platelet quality and function [4, 15, 18, 20, 21]. Recent studies have shown that the integration of PR technology into double‐donation platelet manufacturing can consistently produce high‐quality products that meet European guidelines [10, 12, 22]. However, for the successful implementation of this combined system, it is critical that platelet production is optimized to ensure compatibility with existing automated processing systems and PR platforms, operational efficiency and full compliance with regulatory standards [10, 16, 22].

The present study describes, for the first time, the combination of PR technology with automated pooling methods for creating DD pools. The operational challenge of automated production was addressed by combining the TACSI® system with INTERCEPT to produce pathogen‐reduced double‐dose (PRDD) platelet units. The TACSI® system is a fully automated platform that integrates pooling, centrifugation, separation and leukoreduction into a single streamlined process [23]. It enables efficient and consistent production of pooled PCs, improving quality and reducing manual variability [13, 24]. While TACSI® is widely used for standard pooled platelet production, its application in the context of pathogen inactivation has not been comprehensively studied or validated.

The TACSI® system, when used with the optimized program outlined in the present study, produced pooled products meeting all INTERCEPT entry requirements, including volume, platelet yield, leukocyte content and plasma ratio. Furthermore, all PR SD units generated from these pools met EDQM quality specifications, demonstrating excellent reproducibility and process robustness. These findings validate the use of TACSI® for PR‐compatible DD pools and support its role in optimizing platelet component manufacturing in high‐throughput environments [13, 23, 24, 25].

While the use of 7‐BC pooling for DD preparation minimizes manufacturing variability and improves production efficiency, it could also modestly increase donor exposure compared to traditional four to five BC pools [26]. The potential impact on donor exposure should be carefully assessed and risk‐managed in accordance with clinical and regulatory standards [11]. However, the application of INTERCEPT technology to BC platelets effectively mitigates the risks associated with exposure to multiple donors, thereby enhancing transfusion safety [12, 21, 27].

While PR technology proactively enhances transfusion safety, it is important to acknowledge that bacterial screening of PCs, as currently adopted by several blood services, has also proven effective at ensuring product safety, with no confirmed septic transfusion reactions reported [28]. Nevertheless, PR adds a complementary layer of protection by actively inactivating pathogens, addressing potential window‐period infections or undetected contaminants [2, 4].

The in vitro storage study further demonstrated that PR‐treated platelets retained acceptable quality and function over 8 days of storage. Although a gradual decline in platelet concentration was observed, the trend was consistent with known storage lesion patterns and remained within regulatory thresholds [29, 30]. In vitro platelet functionality, assessed through CD62P expression and TRAP responsiveness, showed the expected increase in activation during storage in both groups. Although CD62P expression was generally comparable between groups, a slight difference was observed: untreated platelets showed higher activation up to Day 2, while PR‐treated platelets had higher activation towards the end of storage. This difference was small and likely influenced by the small sample size, consistent with previous studies that reported small or no differences in CD62P expression [15, 30, 31].

A modest reduction in TRAP responsiveness was detected in PR‐treated platelets compared to untreated controls, which is in agreement with previous studies reporting reduced activation responses following amotosalen/UVA PR [21, 31]. Nevertheless, PR‐treated platelets maintained high levels of responsiveness (>80%) throughout storage, supporting their preserved functional capacity and viability during shelf‐life. Notably, PS exposure remained low and comparable between groups, indicating that PR did not increase apoptotic signalling or compromise platelet membrane integrity [31]. Maximum expression of Annexin V–PS binding was observed in both groups, suggesting in vitro enhanced platelet procoagulant activity [32, 33].

We found a significant increase in glucose consumption over time in both PR‐treated and untreated platelets, highlighting the expected metabolic activation during platelet storage [34]. However, in contrast to previous studies that reported enhanced metabolic activity in PR‐treated platelets [15, 29, 35], our data show that PR‐treated platelets consistently maintained higher glucose reserves than untreated units, suggesting reduced metabolic activity in PR‐treated platelets. This outcome may reflect the optimized processing conditions, including the use of the TACSI system, upstream leukoreduction, CAD incubation time and new gas‐permeable containers, which likely mitigated metabolic stress. This supports previous findings that process parameters play a critical role in preserving platelet metabolism under controlled PR conditions [10, 36, 37].

The comparison of PR‐treated and control units until expiry at Day 8 was done using unsampled split components. This study design avoided any repeated sampling effect and enabled a direct comparison of the final quality parameters. Platelet counts, metabolic markers and functional assays were comparable between groups, supporting the preservation of platelet quality after INTERCEPT treatment and the feasibility of extending shelf‐life to Day 8. It is acknowledged that the results described in the present study are based on a small sample size, which might impact the statistical power of our findings. However, taken together, both the validation and optimization processes involved a larger number of DD pools, generating very reliable results and strengthening the overall robustness of our conclusions.

This study demonstrates that the combination of TACSI® pooling and INTERCEPT PR can produce high‐quality DD platelet components with preserved in vitro quality and functionality through 8 days of storage. The optimized process is reproducible and scalable and aligns with modern transfusion practice goals, thereby enhancing safety while improving operational efficiency. This innovative approach provides a strong foundation for future implementation in routine manufacturing of DD PR platelet components.

## CONFLICT OF INTEREST STATEMENT

The authors declare no conflicts of interest.

## Data Availability

Data are available following a specific request to research@ibts.ie and in line with the Irish Blood Transfusion Service data protection policy and the European General Data Protection Regulations.

## References

[1] Stormer M , Vollmer T . Diagnostic methods for platelet bacteria screening: current status and developments. Transfus Med Hemother. 2014;41:19–27. 24659944 10.1159/000357651 PMC3949613

[2] Levy JH , Neal MD , Herman JH . Bacterial contamination of platelets for transfusion: strategies for prevention. Crit Care. 2018;22:271. 30367640 10.1186/s13054-018-2212-9 PMC6204059

[3] Domanovic D , Ushiro‐Lumb I , Compernolle V , Brusin S , Funk M , Gallian P , et al. Pathogen reduction of blood components during outbreaks of infectious diseases in the European Union: an expert opinion from the European Centre for Disease Prevention and Control consultation meeting. Blood Transfus. 2019;17:433–448. 31846608 10.2450/2019.0288-19 PMC6917531

[4] Irsch J , Lin L . Pathogen inactivation of platelet and plasma blood components for transfusion using the INTERCEPT Blood System. Transfus Med Hemother. 2011;38:19–31. 21779203 10.1159/000323937 PMC3132977

[5] Lin L , Hanson CV , Alter HJ , Jauvin V , Bernard KA , Murthy KK , et al. Inactivation of viruses in platelet concentrates by photochemical treatment with amotosalen and long‐wavelength ultraviolet light. Transfusion. 2005;45:580–590. 15819680 10.1111/j.0041-1132.2005.04316.x PMC7202329

[6] Corash L . Pathogen reduction technology: methods, status of clinical trials, and future prospects. Curr Hematol Rep. 2003;2:495–502. 14561394

[7] Mundt JM , Rouse L , Van den Bossche J , Goodrich RP . Chemical and biological mechanisms of pathogen reduction technologies. Photochem Photobiol. 2014;90:957–964. 25041351 10.1111/php.12311 PMC4277684

[8] Janetzko K , Lin L , Eichler H , Mayaudon V , Flament J , Kluter H . Implementation of the INTERCEPT Blood System for platelets into routine blood bank manufacturing procedures: evaluation of apheresis platelets. Vox Sang. 2004;86:239–245. 15144528 10.1111/j.0042-9007.2004.00419.x

[9] Lu W , Fung M . Platelets treated with pathogen reduction technology: current status and future direction. F1000Res. 2020;9:F1000. 10.12688/f1000research.20816.1 PMC6979468 32047608

[10] Ohlsson S , Diedrich B , Uhlin M , Sandgren P . Optimized processing for pathogen inactivation of double‐dose buffy‐coat platelet concentrates: maintained in vitro quality over 7‐day storage. Vox Sang. 2018;113:611–621. 30156292 10.1111/vox.12696

[11] Abedi MR , Doverud AC . Preparation and pathogen inactivation of double dose buffy coat platelet products using the INTERCEPT blood system. J Vis Exp. 2012;70:e4414. 10.3791/4414 PMC3607280 23242463

[12] Rosskopf K , Helmberg W , Schlenke P . Pathogen reduction of double‐dose platelet concentrates from pools of eight buffy coats: product quality, safety, and economic aspects. Transfusion. 2020;60:2058–2066. 32619068 10.1111/trf.15926 PMC7540585

[13] Cid J , Magnano L , Lozano M . Automation of blood component preparation from whole blood collections. Vox Sang. 2014;107:10–18. 24446859 10.1111/vox.12131

[14] Kumar R , Dhawan HK , Sharma RR , Kaur J . Buffy coat pooled platelet concentrate: a new age platelet component. Asian J Transfus Sci. 2021;15:125–132. 34908742 10.4103/ajts.AJTS_72_19 PMC8628246

[15] Sandgren P , Diedrich B . Pathogen inactivation of double‐dose buffy‐coat platelet concentrates photochemically treated with amotosalen and UVA light: preservation of in vitro function. Vox Sang. 2015;108:340–349. 25557354 10.1111/vox.12232

[16] Perez Aliaga AI , Labata G , Aranda A , Cardoso M , Puente F , Domingo JM , et al. Improvement of blood processing and safety by automation and pathogen reduction technology. Transfus Med Hemother. 2021;48:290–297. 34803572 10.1159/000516696 PMC8578802

[17] European Directorate for the Quality of Medicines & HealthCare . Guide to the preparation, use and quality assurance of blood components. 22nd ed. Strasbourg: Council of Europe; 2025.

[18] Butler C , Doree C , Estcourt LJ , Trivella M , Hopewell S , Brunskill SJ , et al. Pathogen‐reduced platelets for the prevention of bleeding. Cochrane Database Syst Rev. 2013;3:CD009072. 10.1002/14651858.CD009072.pub2 23543569

[19] Rebulla P , Prati D . Pathogen reduction for platelets‐a review of recent implementation strategies. Pathogens. 2022;11:142. 35215085 10.3390/pathogens11020142 PMC8879285

[20] Ladaique P , Etienne JM , Pedini P , Chiaroni J , Vey N , Picard C , et al. Therapeutic efficacy of platelet transfusion treated with amotosalen/UVA pathogen inactivation technology (INTERCEPT(TM) Blood System) in acute myeloid leukemia patients undergoing chemotherapy with curative intent: a single center experience. Blood Transfus. 2023;21:400–408. 36795348 10.2450/2023.0143-22 PMC10497385

[21] McCullough J , Vesole DH , Benjamin RJ , Slichter SJ , Pineda A , Snyder E , et al. Therapeutic efficacy and safety of platelets treated with a photochemical process for pathogen inactivation: the SPRINT Trial. Blood. 2004;104:1534–1541. 15138160 10.1182/blood-2003-12-4443

[22] Amato M , Seekircher L , Tschiderer L , Willeit P , Schennach H , Siller A . Optimized protocol for producing pathogen‐inactivated double‐dose platelet concentrates from six pooled Buffy coats. Ann Lab Med. 2025. Epub ahead of print. 10.3343/alm.2024.0555 PMC12535830 40114655

[23] Blood T , Technologies C . TACSI System – Improve efficiency by processing up to six pooled buffy coats at once. Available from https://www.terumobct.com/en/gl/products-services/global-blood-solutions/global-blood-solutions-products/tacsi.html. Last Accessed 17 Jul 2025

[24] Sandgren P , Hild M , Sjodin A , Gulliksson H . Storage of Buffy‐coat‐derived platelets in additive solutions: in vitro effects on platelets prepared by the novel TACSI system and stored in plastic containers with different gas permeability. Vox Sang. 2010;99:341–347. 20576025 10.1111/j.1423-0410.2010.01364.x

[25] Plaza EM , Cespedes P , Fernandez H , Sanchez‐Guiu MI , Egea JM , Vicente V , et al. Quality assessment of buffy‐coat‐derived leucodepleted platelet concentrates in PAS‐plasma, prepared by the OrbiSac or TACSI automated system. Vox Sang. 2014;106:38–44. 23888911 10.1111/vox.12072

[26] van der Meer P . Apheresis versus whole‐blood‐derived platelets: pros and cons. ISBT Sci Ser. 2012;7:112–116.

[27] van Rhenen D , Gulliksson H , Cazenave JP , Pamphilon D , Ljungman P , Kluter H , et al. Transfusion of pooled buffy coat platelet components prepared with photochemical pathogen inactivation treatment: the euroSPRITE trial. Blood. 2003;101:2426–2433. 12456508 10.1182/blood-2002-03-0932

[28] O'Flaherty N , Bryce L , Nolan J , Lambert M . Changing strategies for the detection of bacteria in platelet components in Ireland: from primary and secondary culture (2010–2020) to large volume delayed sampling (2020–2023). Microorganisms. 2023;11:2765. 38004776 10.3390/microorganisms11112765 PMC10673373

[29] Escolar G , Diaz‐Ricart M , McCullough J . Impact of different pathogen reduction technologies on the biochemistry, function, and clinical effectiveness of platelet concentrates: an updated view during a pandemic. Transfusion. 2022;62:227–246. 34870335 10.1111/trf.16747 PMC9300014

[30] Lozano M , Galan A , Mazzara R , Corash L , Escolar G . Leukoreduced buffy coat‐derived platelet concentrates photochemically treated with amotosalen HCl and ultraviolet A light stored up to 7 days: assessment of hemostatic function under flow conditions. Transfusion. 2007;47:666–671. 17381625 10.1111/j.1537-2995.2007.01169.x

[31] Abonnenc M , Sonego G , Kaiser‐Guignard J , Crettaz D , Prudent M , Tissot JD , et al. In vitro evaluation of pathogen‐inactivated buffy coat‐derived platelet concentrates during storage: psoralen‐based photochemical treatment step‐by‐step. Blood Transfus. 2015;13:255–264. 25369598 10.2450/2014.0082-14 PMC4385074

[32] Escolar G , McCullough J . Platelet in vitro assays: their correspondence with their in vivo hemostatic potential. Transfusion. 2019;59:3783–3793. 31642072 10.1111/trf.15559

[33] Reddy EC , Rand ML . Procoagulant phosphatidylserine‐exposing platelets in vitro and in vivo. Front Cardiovasc Med. 2020;7:15. 32195268 10.3389/fcvm.2020.00015 PMC7062866

[34] Aibibula M , Naseem KM , Sturmey RG . Glucose metabolism and metabolic flexibility in blood platelets. J Thromb Haemost. 2018;16:2300–2314. 30151891 10.1111/jth.14274

[35] Johnson L , Loh YS , Kwok M , Marks DC . In vitro assessment of buffy‐coat derived platelet components suspended in SSP+ treated with the INTERCEPT Blood system. Transfus Med. 2013;23:121–129. 23480103 10.1111/tme.12020

[36] Sandgren P . Preserved in vitro metabolic and functional characteristics of double‐dose apheresis platelet concentrates photochemically treated with amotosalen and ultraviolet a light. Blood Transfus. 2018;16:118–120. 27723448 10.2450/2016.0117-16 PMC5770323

[37] Lotens A , de Valensart N , Najdovski T , Acquart S , Cognasse F , Rapaille A . Influence of platelet preparation techniques on in vitro storage quality after psoralen‐based photochemical treatment using new processing sets for triple‐dose units. Transfusion. 2018;58:2942–2951. 30362131 10.1111/trf.14909

